# Short–Term Exposure to Electromagnetic Fields Generated by Mobile Phone Jammers Decreases the Fasting Blood Sugar in Adult Male Rats

**Published:** 2016-03-01

**Authors:** F. Shekoohi Shooli, S. A. R. Mortazavi, S. Jarideh, S. Nematollahii, F. Yousefi, M. Haghani, S. M. J. Mortazavi, M. B. Shojaei-fard

**Affiliations:** 1 Ionizing and Non-ionizing Radiation Protection Research Center (INIRPRC), Shiraz University of Medical Sciences, Shiraz, Iran; 2 Student Research Committee, School of Medicine, Shiraz University of Medical Sciences, Shiraz, Iran; 3 Professor of Medical Physics, Medical Physics Department, School of Medicine, Shiraz University of Medical Sciences, Shiraz, Iran; 4 Department of Physiology, Fasa University of Medical Sciences, Fasa, Iran

**Keywords:** Electromagnetic fields, Mobile jammer, Fasting blood sugar

## Abstract

**Background:**

Substantial evidence indicates that exposure to electromagnetic fields (EMF) above certain levels can affect human health through triggering some biological responses. According to WHO, short-term exposure to EMF at the levels present in the home/environment do not cause any apparent detrimental effects in healthy individuals. However, now, there is a debate on whether long-term exposure to low level EMF can evoke detrimental biological responses. Although based on the Communications Act of 1934, selling, advertising, using, or importing mobile jammers which block cell phone calls and text messages are illegal acts, in some countries these devices are being used for security purpose and for prevention of cheating during examinations.

**Methods:**

In this study 30 male Wistar rats were randomly divided into 3 groups of 10 each. The control group received no radiation. The sham exposure group was exposed to a switched-off jammer device. After fasting for 12 hours, the exposure group was exposed to EMFs at a distance of 50 cm from the jammer. Blood samples were collected from the tail vein after 24, 48 and72 hours and fasting blood sugar was measured by using a common blood glucose monitor (BIONIME GM110, Taiwan). The significance level was considered 5% and SPSS Ver. 21 was used for statistical analysis. The data were analyzed by ANOVA followed by Tukey’s test.

**Results:**

A statistically significant difference was observed between blood sugar level in the control and exposure groups after 24, 48 and 72 hours of continuous irradiation (p values were <0.001, <0.001 and 0.002, respectively). No significant difference was found between the level of fasting blood sugar in control and sham groups.

**Conclusion:**

Short-term exposure to electromagnetic field generated by mobile phone jammer can reduce blood sugar level in adult male rats. These findings, in contrast with our previous results, lead us to this conclusion that the use of these signal blocking devices in very specific circumstances may have some therapeutic effects. However, further studies have to be performed to find out the exact mechanism by which Jammer EMFs reduce fasting blood sugar.

## Introduction

Nowadays, mobile phones are widely used in our daily life. The broad use of cell phones has led to some global concerns about the safety of these devices. Mobile phone use may be problematic in some cases. The solution for this problem is using mobile phone jammer devices which block cell phones. These devices are known as cell phone jammers or “GSM jammers”[1]. A GSM jammer is a device that transmits signals at the same frequency as GSM mobile signals. Therefore, cell phones can be inactivated in the area where the jammer system is on.  All phones within the effective radius of the jammer will be blocked. Also the basic parameter of the distance from the phone is very important since the amount of the output power of the jammer depends on the area where we need to jam the signals[2].

In recent years, the effect of electromagnetic fields generated by mobile phones[3-10], mobile phone base stations[11], mobile phone Jammers[12] , Laptop computers[13] Radars[4] and MRI[14] on the health of humans and animals have been studied in our researches center. The results of these studies have shown that electromagnetic fields can reduce the reaction time of students exposed to mobile phone radiations for 10 minutes[5]. Exposure to electromagnetic fields can release mercury from dental amalgam[9, 14], decrease sperm quality and motility[13], and also decrease the level of thyroid hormones and TSH[8]. Electromagnetic field of cell phones can cause some changes in the level of hormones such as insulin and plasma lipids as well as blood sugar[15]. It can also decrease the total antioxidant capacity[15-17]. Short-term exposure to electromagnetic field of cell phones can affect the energy metabolism of the brain[18].Electromagnetic field emitted from mobile phones can produce impairments in some biochemical phenomena and oxidative stress in brain, liver and renal tissue of rat[19]. On the other hand, the use of signal blocking jammers has led to some concerns about the adverse health effects of these devices. In many countries, marketing of these devices is forbidden[20]. However in other countries, these devices can be used in exam halls at schools and universities. In this paper, the effect of Jammer system on fasting blood glucose level in male rats is studied.

## Material and Methods

The jammer (model MB06) used in this study was capable of blocking mobile communications within distances ranged up to 40 meters. Thirty male rats, weighing 200-250 g, were randomly divided into three groups of 10 each; consisting the control, sham (exposed to a switched off jammer device), and case group (exposure to jammer radiation). Case group were placed in restrainers, at the distance of 50 cm from the Jammer, for 24, 48 and 72 hours (continuous irradiation). The animals were maintained in a standard environmental condition and were fed with standard diet and tap water. All animal experiments were approved by the animal experiment ethics committee of the Shiraz University of Medical Sciences.  The animals were deprived of food for 12 hours since 6 PM until 6 AM in the next morning. Blood sampling was performed from tail vein at 8 AM. All statistical tests were carried out at the significance level of 0.05 using SPSS version 21. The data were analyzed by ANOVA followed by Tukey test. 

## Results

In this study, there was a significant difference between fasting blood sugar level in the control and case groups after 24 (P value = 0.001), 48 (P value = 0.00001) and 72 (P value = 0.002) hours of continuous irradiation by a Jammer device. However, there were no significant differences between the level of fasting blood sugar in control and sham groups in adult male rats. Furthermore, the duration of exposure was not linked to the level of fasting blood sugar. Table 1 shows the mean fasting blood sugar in control, exposed and sham-exposed groups 24, 48 and 72 hours after exposure to jammer microwave radiation.

**Table 1:** Mean fasting blood sugar level in different groups at the beginning of study and 24, 48 and 72 hours after exposure to radiation in both active and inactive jammer

	**Sample Size**	**Fasting Blood Sugar Level ** **(Mean SD)**				**Sig (P-value)**
		**0 h**	**24 h**	**48 h**	**72 h**	
Control	10	92.2±13.41	92.2±13.41	92.2±13.41	92.2±13.41	> 0.99
Sham	10	92.2±13.41	96.0±12.99	72.7±13.79	105.5±14.15	0.106
Exposed	10	92.2±13.41	76.9±8.42	75.3±6.71	75.6±12.39	0.935
Sig (P-value)		> 0.99	0.001	0.001	0.001	

## Discussion

The effects of exposure to electromagnetic fields produced by activated mobile phones on fasting blood glucose have been studied before. Meo and Rubeaan showed that rats exposed to mobile phone radiation for durations longer than 15 min/day for 3 months revealed a significant increased fasting blood glucose level compared to the control group. These researchers concluded that long-term exposure to activated mobile phone can be associated with the increase in fasting blood glucose in rats[21]. Havas reported that extremely low-frequency (ELF) radiation at longer periods of exposure significantly increased fasting blood sugar level in EMF exposure group compared to control group. These results showed that the plasma glucose level, in response to electromagnetic pollution (dirty electricity), increased Type 2 diabetic patients[22]. Similar results were reported by Rajendra et al. They found elevated levels of norepinephrine in the brain of fertilized chick eggs on day 15 of exposure to 5, 50, and 100 µT[23]. Li et al. also in their in vitro study, exposed hepatocytes to 50 Hz pulsed electric fields (0.7V/m). They reported a conformation change in the insulin molecule in the exposed group compared to control group[24]. Jolley et al. exposed Langerhans islets of rabbits to low frequency pulsed electromagnetic field and found a decrease in insulin release during glucose stimulation, compared to control group[25]. Similarly, Navakatikyan et al. exposed rats to 50Hz magnetic fields 23 hours per day for 11 days at 10, 50, and 250 µT. The serum insulin level decreased at the middle- and high-flux densities, this alteration was supposed to be linked to stress by the authors[26]. Changes in glucose level as a response to EM radiation (Exposure to electromagnetic pollution) in different doses may lead to misdiagnosis of diabetes. Reducing the exposure to electromagnetic pollution by avoiding jammer devices or using specially designed GS filters may enable some diabetics to better regulate their blood sugar with less medication and also help borderline or pre-diabetics to remain non diabetic for a longer time. In our study after 24, 48 and 72 hours of exposure to jammer, fasting blood sugar level significantly reduced in case group compare to that of control group.

The result of this study is not in line with the findings of other researchers who reported increased glucose level after exposure to electromagnetic field. This inconsistency may be due to different durations of exposure to EMF; which was a short-term (24-72 h) exposure in our study, while it was long term (10-90 days) exposure in other studies. It has been shown that binding capacity of insulin to its receptors was reduced 87% after exposure to pulsed electric fields, which leads to increased blood sugar[24]. Based on our results, it seems that this process doesn’t occur in short-term exposures. Mortazavi et al. showed that 10 minutes exposure to mobile phone electromagnetic fields decreased computer-assisted visual reaction time, which may indicate increased brain activity[5]. In another study, Volkow et al. showed that 50 minutes exposure to cell phone electromagnetic fields was associated with increased brain glucose metabolism in the regions that were close to the antenna[27]. Generally, about 25% of blood glucose consumption is due to brain activity, and increased brain activity due to exposure to electromagnetic fields may cause increased brain glucose consumption and decreased blood glucose levels. However, future evaluation is needed to approve these finding.

## Conclusion

Short-term exposure to electromagnetic fields of jammer device leads to decreased blood glucose level, which may occur via increased brain activity and therefore increased brain glucose metabolism. 
